# Nebulized mesenchymal stem cell derived conditioned medium ameliorates *Escherichia coli* induced pneumonia in a rat model

**DOI:** 10.3389/fmed.2023.1162615

**Published:** 2023-06-02

**Authors:** Héctor E. González, Sean D. McCarthy, Claire Masterson, John G. Laffey, Ronan MacLoughlin, Daniel O’Toole

**Affiliations:** ^1^REMEDI at CÚRAM Medical Devices Center and Discipline of Anesthesia, University of Galway, Galway, Ireland; ^2^Aerogen Ltd., Galway, Ireland

**Keywords:** mesenchymal stem cell, pneumonia, secretome, nebulizer, aerosol—therapeutic

## Abstract

**Background:**

Mesenchymal stem cells (MSC) have shown immense therapeutic promise in a range of inflammatory diseases, including acute respiratory distress syndrome (ARDS), and are rapidly advancing through clinical trials. Among their multimodal mechanisms of action, MSCs exert strong immunomodulatory effects via their secretome, which contains cytokines, small molecules, extracellular vesicles, and a range of other factors. Recent studies have shown that the MSC secretome can recapitulate many of the beneficial effects of the MSC itself. We aimed to determine the therapeutic capacity of the MSC secretome in a rat bacterial pneumonia model, especially when delivered directly to the lung by nebulization which is a technique more appropriate for the ventilated patient.

**Methods:**

Conditioned medium (CM) was generated from human bone marrow derived MSCs in the absence of antibiotics and serum supplements. Post-nebulization lung penetration was estimated through nebulization of CM to a cascade impactor and simulated lung and quantification of collected total protein and IL-8 cytokine. Control and nebulized CM was added to a variety of lung cell culture models and injury resolution assessed. In a rat *E. coli* pneumonia model, CM was instilled or administered by nebulization and lung injury and inflammation assessed at 48 h.

**Results:**

MSC-CM was predicted to have good distal lung penetration and delivery when administered by nebulizer. Both control and nebulized CM reduced NF-κB activation and inflammatory cytokine production in lung cell culture, while promoting cell viability and would closure in oxidative stress and scratch wound models. In a rat bacterial pneumonia model, both instilled and nebulizer delivered CM improved lung function, increasing blood oxygenation and reducing carbon dioxide levels compared to unconditioned medium controls. A reduction in bacterial load was also observed in both treatment groups. Inflammatory cytokines were reduced significantly by both liquid and aerosol CM administration, with less IL-1β, IL-6, and CINC1 in these groups compared to controls.

**Conclusion:**

MSC-CM is a potential therapeutic for pneumonia ARDS, and administration is compatible with vibrating mesh nebulization.

## Introduction

Acute respiratory distress syndrome (ARDS) is a severe disease of the lung with a continuing high mortality. It is diagnosed on the basis of acute onset not attributable to background disease, infiltration of blood or tissue derived cells to the airspace and impaired blood oxygenation levels, the last of which can be used to denote severity level (1). The most problematic form of ARDS arises from infectious etiologies, commonly as a result of bacterial or viral infection, termed pneumonia (2). Here the infectious particles trigger an immune response in the lung epithelium and resident alveolar macrophages causing the production of a wide range of soluble signaling factors termed the “cytokine storm,” which in turn signals to a wide a variety of tissue and immune effector cells (3). Inflammation and fluid in the airspace eventually impair gas exchange resulting in hypoxemia and possible death. Current standard of care is largely supportive, with hydration, immediate broad-spectrum antibiotics and mechanical ventilation continuing to be mainstays of therapy (4). Of particular concern is the rise in antimicrobial resistant (AMR) strains of bacteria which mean more reliance on the patient’s own immune system to clear the infection, which may not be sufficient. Despite many promising preclinical tests, an effective specific therapy for ARDS remains elusive.

Mesenchymal stem/stromal cells (MSCs) have recently risen to prominence in the field of inflammation therapeutics (5). Their multimodal immunomodulatory action has led to investigations in the fields of graft versus host disease (GvHD), diabetic ulcers, critical limb ischemia, and acute kidney injury, to name but a few (6–8). They have also been widely developed for both chronic and acute lung diseases, with promising *in vitro* and preclinical results now graduating to clinical trials (9). While bone marrow is the most studied tissue source of MSCs, they have been prepared from a wide range of tissues by a multiplicity of isolation techniques. However, several issues of practicality have emerged regarding stem cell therapy, including difficulty and expense of human dose manufacture, the requirement of careful cryogenic preservation during storage and transport, and lingering concerns regarding the introduction of a foreign living cell to the patient (10, 11). To alleviate these concerns, recent studies have shown that various MSC products can recapitulate many of the beneficial activities of the MSC itself, including in lung disease model settings (12). While many groups have investigated specific secreted factors such as extracellular vesicles (EV), the total secretome or “conditioned medium” (CM) is also an attractive option for inflammatory, antibacterial and other research and is readily available as a by-product of the MSC manufacturing process.

In light of these emerging possibly superior therapeutic options, our overall hypothesis is that MSC-CM will replicate previous findings with MSC and MSC-EVs in ARDS models and prove a more abundant, more accessible and less expensive source of medicine. We have aimed to build on previous *in vitro* work to apply MSC-CM to cell and animal models of pneumonia. We have also aimed to utilize the novel administration option of vibrating mesh nebulization to the airspace, which is a technique that requires a smaller dose and is likely to produce a stronger and faster effect as it is delivered directly to the affected organ. Of relevance, delivery of MSC themselves intratracheally has previously been shown to be effective in an ARDS model (13, 14), although delivery of volumes of liquid suspensions to the lungs is not considered a viable approach in patients due to an existing dangerous accumulation of fluid. Vibrating mesh nebulization has been previously described to preserve the activity of a broad range of proteins and other soluble factors due the low shear stress applied on solutions during aerosolization, yet it is also capable of providing a mist that penetrates to the distal lung under mechanical ventilation which is the situation in ARDS (15–18).

## Methods

### Mesenchymal stem cell conditioned medium

Human bone marrow derived MSCs were isolated from healthy donor tissue based on plastic adherence. Briefly, after obtaining informed consent, bone marrow aspirate was sampled from the iliac crest, diluted with minimum essential media alpha (MEMα; Sigma Aldrich, Arklow, Ireland) containing 10% fetal bovine serum (FBS; Gibco, ThermoFisher Scientific, Waltham, MA, United States) and penicillin/streptomycin, and plated to tissue culture flasks (Corning Limited, Dublin, Ireland) at 10^6^ cell per cm^2^. After 24 h in a humidified incubator at 37°C and 5% CO_2_ in air, non-adherent cells were aspirated and remaining cells washed with phosphate buffered saline (PBS). After a further 7 days of incubation with medium refresh on the third day, MSC colonies were trypsinized, counted, and cryopreserved as passage 0. MSC markers were confirmed by flow cytometry as per the minimum criteria for MSC established by Dominici et al. (data not shown) (19). Cryovials of MSCs were then seeded to tissue culture plastic and expanded to 175 cm^2^ flasks at passage 3. At 80% confluence, cells were treated with either lipopolysaccharide (LPS) for nebulizer dynamics studies or vehicle for other studies. After 24 h, medium was aspirated, cells washed with PBS and flasks refed with 15 mL of MEMα with no additions, i.e., serum free medium (SFM). After 24 h of further incubation, conditioned medium (CM) was aspirated, centrifuged at 1,000 × *g* for 10 min to remove cells and cell debris. After that, CM was transferred into centrifugal concentrator units (Amicon® Ultra −15 Centrifugal filter Units) with 3 KDa MWCO limit size membrane. Tubes were places in a centrifuge (Epperndorf™ 5810R, Epperndorf, Hamburg, Germany) with swinging rotor at 4,000 × *g* at 4°C until desired final volume. In addition, concentrated CM was transferred into dialysis tubes (Tube-O DIALYZER™ mini dialysis system) with a pore size of 4 KDa MWCO. Dialyzer tubes were placed in closed buckets and exposed to PBS with a ratio of 500 mL of PBS per 100 μL of concentrated CM for 24 h at 4°C. The CM was then collected, aliquoted, and stored at −80°C.

### Nebulization and collection of CM

For all experiments where control and nebulized CM were to be compared, in a tissue culture cabinet a vibrating mesh nebulizer (VMN; Aerogen Solo®; Aerogen, Galway, Ireland) was placed so that the lower part fitted into a 50 mL polypropylene tube and the contact points sealed with Parafilm. The controller box was turned on until the CM was passed through and condensed in the tube and a centrifugation (4,000 × *g*, 5 min) settled all the liquid to the bottom. This process took c. 1 min per mL of CM and recovery was c. 95% by volume.

### Nebulized conditioned medium aerosol dynamics

10 mL of LPS-induced CM (LPS-CM) was defrosted, nebulized, and collected. The nebulizer was then washed with PBS and then inverted and washed again with PBS with aerosol collected each time. 10 mL of LPS-CM was then nebulized to a cascade impactor [Next Generation Impactor (NGI); Copley Scientific Ltd., Colwick, United Kingdom] under a normal breathing ventilation regimen (15 L per minute) and each collection plate and connector washed with 10 mL of PBS and collected. Finally, 10 mL of LPS-CM was applied to a simulated lung device (ASL 5000™ Lung Solution; Laerdal, Wappingers Falls, NY, United States), again under normal breathing ventilation, and the various collection filters washed with 10 mL PBS and collected. All samples were then analyzed for total protein content by BCA assay (Pierce BCA Protein Assay Kit; Thermofisher Scientific) and a representative cytokine quantified by ELISA as per manufacturer’s instructions (Human IL-8/CXCL8 DuoSet; R&D Systems, Minneapolis, MN, United States).

### *In vitro* lung epithelial model studies

An A549 lung epithelial cell line incorporating an NF-κB driven luciferase reporter construct (A549-κB-luc; Panomics, Fremont, CA, United States) was used for transcription factor assessment and scratch wound assays. A BEAS2B bronchial epithelial cell line (ATCC, Manassas, VA, United States) was used for other injury models. These were both cultured in RPMI-1640 (Sigma) with 10% FBS and penicillin/streptomycin. *Reporter assays:* A549-κB-luc cells were seeded to 96 well plates at 50,000 cells in 100 μL of medium per well and 50 μL of SFM, non-nebulized or nebulized and collected MSC-CM was added 24 h later. An inflammatory activation cocktail (IL-1β 10 ng/mL, TNF-α 50 ng/mL, and IFN-γ 50 ng/mL) was then added for a further 24 h, whereupon medium was aspirated, cells homogenized in luciferase substrate (Bright-Glo™ Luciferase Assay System; Promega, Madison, WI, United States) and luminescence quantified in a Victor™ multilabel plate reader (PerkinElmer, Waltham, MA, United States). *Scratch wound assays:* A549-κB-luc cells were seeded to 24 well plates at 200,000 cells in 1 mL of medium. When confluence was reached, a single scratch wound was performed on the cell monolayer using a 1,000 μL pipette tip, medium aspirated, and cells washed with PBS. 1 mL of fresh medium was then added to each well with 250 μL of SFM, non-nebulized or nebulized MSC-CM. At various timepoints up to 24 h, scratch wounds were imaged under light microscopy (Cytation 5™; BioTek, Shoreline, WA, United States) and total remaining wound size quantified. *Endotoxin inflammation model:* BEAS2B were seeded to 96 well plates at 50,000 cells in 100 μL of medium per well and 50 μL of SFM, non-nebulized or nebulized and collected MSC-CM was added 24 h later. *Escherichia coli* derived lipopolysaccharide (LPS; Sigma) was then added to each well at 1 μg/mL. After another 24 h, media was harvested for cytokine ELISA (Human IL-1β, IL-8/CXCL8 Duoset; R&D Systems) and cell viability assessed through addition of fresh media with 3-(4,5-dimethylthiazol-2-yl)-2,5-diphenyltetrazolium bromide (MTT; Sigma) at a final concentration of 0.5 mg/mL. After 2 h, media was aspirated, formazan crystals solubilized in 100 μL of DMSO, and absorbance read at 630 nm. *Oxidative stress model:* BEAS2B cells were seeded as above and MSC-CM added. Hydrogen peroxide (H_2_O_2_) was then added to a final concentration of 10 mM for 2 h and an MTT viability assays performed as described above.

### *In vitro* antibacterial activity models

*Direct bacterial inhibition:* Overnight bacterial cultures were established from cryofrozen beads in 5 mL of Luria Broth (Fannin, Dublin, Ireland) in an orbital shaker at 37°C, 220RPM, overnight. Bacterial strains used were a patient isolate Gram-negative *E. coli* (Department of Clinical Microbiology, University of Galway, Ireland) and a reference Gram-positive *S. aureus* (#5624; ATCC, Manassas, VA, United States). Serial dilution and plating to agar determined these cultures to be c. 5 × 10^9^ and 10 × 10^9^ CFU/mL, respectively. MSC-CM was either used directly or passed through a VMN and added to 96 well V-bottom plates at 200 μL per well. Bacteria was then added in 10 μL of PBS to give final concentrations of 1,000 or 10,000 CFU/μL. Plates were placed in an orbital shaker at 37°C, 220 RPM for 4 h and then centrifuged with a plate adaptor in a benchtop centrifuge at 2,000 × *g* for 10 min. Medium was aspirated, bacteria pellet resuspended in 200 μL and transferred to a flat bottomed 96 well plate. Absorbance was assessed at 650 nm in a multilabel plate reader (Victor™). A linear relationship between absorbance and CFUs has been previously established for these strains (20). *Phagocytosis assay:* THP-1 monocyte cells were seeded to 96 well plates at 100,000 cells per well and differentiated to macrophages with 1 μg/mL of phorbol myristate acetate (PMA) for 72 h. Wells were aspirated and refed with 100 μL of pre-nebulized or control MSC-CM and LPS (100 ng/mL) added to simulate the pneumonia environment. 24 h later, zymosan particles were added (four particles per cell ratio) and phagocytosis allowed to proceed for 4 h. Wells were then imaged under fluorescent microscopy (Leica Biosystems, Clare, Ireland) with two fluorescent particles in a cell being considered positive for phagocytosis activity.

### *In vivo* pneumonia model

All animal experiments were performed under the ethical approval of the Animal Care in Research Ethical Committee (ACREC) at the University of Galway and the regulatory approval of the Health Products Regulatory Agency (HPRA) Ireland.

Adult male Sprague Dawley rats, 350–450 g, were used (Envigo, London, United Kingdom). Animals were anesthetized with inhalational isoflurane (2% in air) and a bolus of clinically isolated *E. coli* delivered intratracheally. Animals were maintained under anaesthesia for 1 h between bacteria administration and treatment and then given IT instillation or attached to a Flexivent small animal ventilator (SciReq, Montral, Canada) for nebulization. Animals were ventilated with room air with 90 breaths per minute, flow rate 500 cc per minute and duty cycle 30%. 300 μL of MSC-CM was delivered to the animals via VMN IT over the course of approximately 5 min. In a separate group, animals were anesthetized and received 300 μL of MSC-CM by direct instillation IT. All animals were allowed to recover and returned to cages for observation.

48 h later, animals were reanesthetized with medetomidine 1 mg/kg (1 mg/mL Medetor®; Chanelle Veterinary, Ireland) and ketamine 75 mg/kg (100 mg/mL Ketamidor, RicherPharma AG, Austria), and after confirmation of anesthesia intubated and attached to the ventilator. Ventilation regime was 90 breaths per minute, flow rate 500 cc per minute. Animals were tracheostomized and a canula inserted in the coronary artery for continuous blood pressure and heart rate monitoring. Arterial blood for gas and metabolite analysis was also taken from this canula intermittently (ABL 90 Flex Plus; Radiometer Ireland, Dublin, Ireland). After 1 h, cisatracurium besilate (10 mg·kg^−1^·h^−1^; Atricorium besylate, Aspe, Ireland) was administered to induce respiratory paralysis immediately prior to static lung compliance measurement. Animals were then given an anesthetic overdose and sacrificed by exsanguination.

Bronchoalveolar lavage was performed by delivering 5 mL of PBS to the lungs by intratracheal instillation and allowing the fluid to drip into a collection tube. This was repeated twice and all three BAL collections pooled.

### Sample analysis

*Lung wet:dry ratio:* The left lung lobe was separated and weighed. It was then placed in a 37°C oven for 24 h to dehydrate it and reweighed. Wet to dry ratio was then calculated. *Leukocyte differential counts:* 5 mL of BAL was centrifuged at 1,000 × *g* for 10 min and the pelleted cells resuspended in 1 mL of PBS. This was then transferred to a glass slide by centrifugation (Cytospin™; ThermoFisher Scientific), dried and stained with Haema-Gurr for 5 min followed by 5 min of ethanol destain and dried in air. Infiltrating cells were imaged under light microscopy (Leica) and total and neutrophil cell numbers counted. *Cytokine multiplex:* BAL was applied to a bead based cytokine multiplex assay (Bio-Plex Pro™ Rat Cytokine 23-Plex Assay; Accuscience, Naas, Ireland) as per manufacturer’s instructions in a multiplex bead reader (Bio-Plex 200 System; Accuscience). *Lung bacterial load:* BAL was serially diluted and plated to Brilliance UTI Clarity agar plates (Fannin). 24 h later *E.coli* presence was confirmed by color of colonies and CFU counted.

### Statistical analysis

All data were analyzed using GraphPad Prism Software (GraphPad Software, San Diego, CA, United States). Data were tested for normal distribution and significance determined by ANOVA, with *p* < 0.05 considered significant.

## Results

### Nebulized MSC-CM has excellent compatibility with VMN delivery

**Figure 1:**
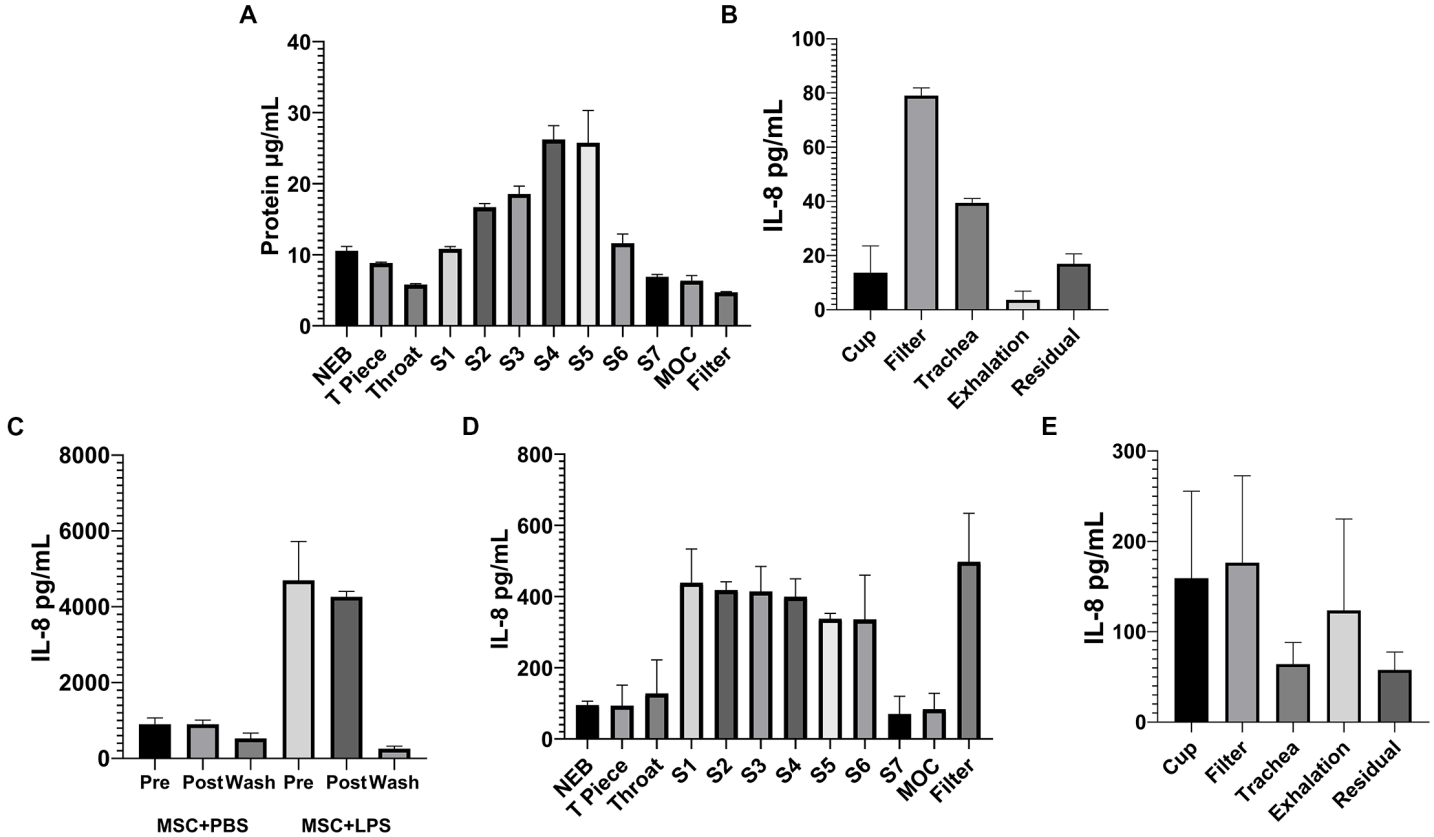
Nebulized MSC-CM will penetrate deep into the lung and has preserved protein content. 10 mL of MSC-CM was applied by VMN to a cascade impactor with an airflow of 15 L/min and protein deposition in the chambers quantified **(A)**. The same MSC-CM was then applied by VMN to a simulated lung and protein deposition to the various parts of the circuit assessed **(B)**. In IL-8 ELISAs as representative of intact protein delivery, naïve and LPS-treated MSC-CM was passed through a VMN and collected **(C)**, and the IL-8 content from the various plates of the cascade impactor **(D)** and points in the simulated lung circuit **(E)** were quantified after delivery of LPS-CM to the devices.

Naïve MSC-CM applied to a cascade impactor by VMN showed delivery to compartments corresponding to airways of diameter 1.39–8.61 mm (Figure 1A) showing deep penetration to the distal lung. Total delivery to a simulated lung was a significant percentage of the sample applied, with 40 μg/mL of protein recovered from the lung compared to only 5 μg/mL in the exhalation filter (Figure 1B), indicating that doses of conditioned medium can be successfully delivered to patient airways. LPS activated MSC-CM contains a high concentration of IL-8 cytokine, which almost completely survived the nebulization process, with little cytokine observed in the filter wash-out (Figure 1C), suggesting that most proteins in the MSC secretome are delivered to the lung intact. However, the profile of IL-8 protein delivered to the airways is somewhat different to that of the total LPS-CM protein (Figures 1D,E). Overall this delivery profile is in line with other aqueous solutions delivered by VMN.

### Vibrating mesh nebulization preserves MSC-CM activity *in vitro*

**Figure 2:**
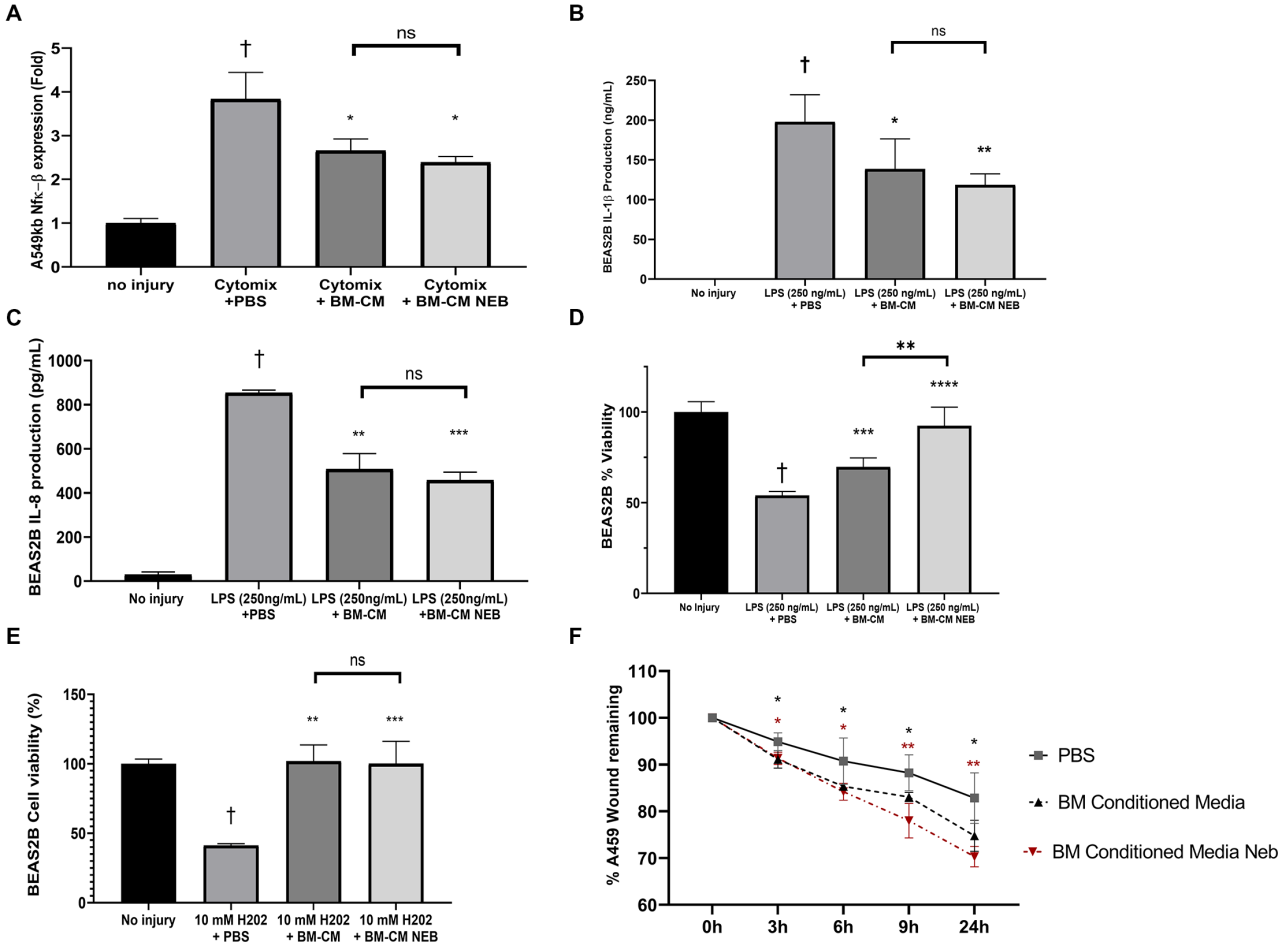
Anti-inflammatory and tissue healing capacity of MSC-CM *in vitro* after nebulization. Nebulization of MSC-CM did not attenuate the induction of the pro-inflammatory pathway NF-κB compared with the non-nebulized CM **(A)**. MSC-CM reduced IL-1β **(B)** and IL-8 **(C)** production while attenuating the decrease in BEAS2B cell viability **(D)** after 250 ng/mL LPS stimulation, with significant increase of effect after nebulization. MSC-CM also attenuated the viability of BEAS2B after oxidative stress damage **(E)** with no reduction in the effect after nebulization. MSC-CM increased wound closure in A549 monolayers at 6, 9, and 24 h after initiation, with no significant reduction in the effect after nebulization (**F**; Data presented mean ± SD. ^†^significant between no injury and PBS group, ^*^significant difference between treated group and PBS group, both *p* ≤ 0.05. ^**^significant difference between treated group and vehicle *p* ≤ 0.01. ^**^significant difference between treated group and vehicle *p* ≤ 0.00.1. NS, no significant difference).

MSC-CM was passed through a VMN, collected, and used in a variety of lung inflammation and injury models. MSC-CM was able to reduce the activity of an NF-κB reporter in alveolar epithelial cells in response to inflammatory cytokine induction and this anti-inflammatory nature persisted after nebulization (Figure 2A). In a bronchial epithelial cell line exposed to *E. coli* LPS induced inflammation, MSC-CM significantly inhibited production of IL-1β (Figure 2B) and IL-8 (Figure 2C) cytokines, and this activity was also preserved after nebulization. This LPS injury model simultaneously resulted in a drop in viability in the bronchial cells, which could be ameliorated by MSC-CM treatment (Figure 2D). Interestingly, nebulized MSC-CM actually performed better than non-nebulized with a significant further increase in cell viability (Figure 2D). In an oxidative stress model, hydrogen peroxide significantly reduced bronchial epithelial cell viability, as measured by MTT assay, and this could be reversed with either control or nebulized MSC-CM (Figure 2E). Finally, in an alveolar epithelial scratch wound model, MSC-CM significantly increased wound closing speed, and pre-nebulization of the MSC-CM actually resulted in an even faster restitution of the epithelial layer (Figure 2F) with representative images available in Supplementary Figure S1.

### Antimicrobial activity of nebulized MSC-CM

**Figure 3:**
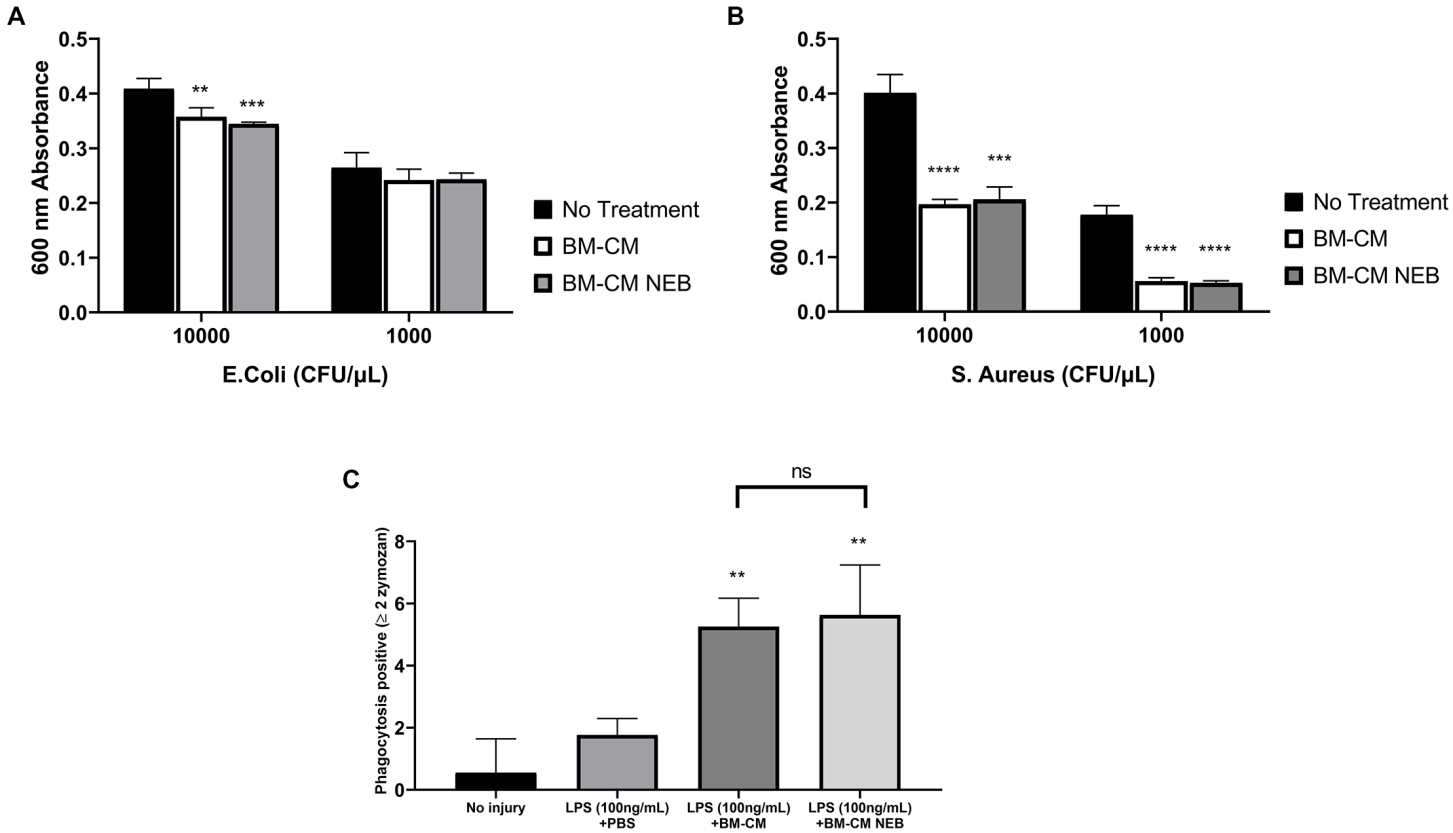
Antibacterial capacity of MSC-CM *in vitro* after nebulization. MSC-CM inhibited the growth of *Escherichia coli* **(A)** and *S. aureus* **(B)** cultures before and after nebulization at an initial bacterial concentration of 10,000 or 1,000 CFU/μL. MSC-CM increased the phagocytic capacity of LPS-stimulated THP-1 cells compared with vehicle treatment **(B)** with no significant decrease in effect after nebulization (Data presented mean ± SD; ^*^significant difference between treated group and no treatment *p* ≤ 0.05; ^**^significant difference between treated group and no treatment *p* ≤ 0.01; ^**^significant difference between treated group and no treatment *p* ≤ 0.00.1; NS, no significant difference).

MSC-CM reduced the proliferation rate of both Gram-negative *E. coli* (Figure 3A) and Gram-positive *S. aureus* (Figure 3B) although this did not reach significance with lower inoculation density of *E. coli* and was particularly pronounced against *S. aureus*. This inhibition was preserved after nebulization in both pathogen strains (Figures 3A,B). In monocyte/macrophages, phagocytosis was enhanced by exposure to both control and nebulized MSC-CM (Figure 3C).

### Nebulized MSC-CM ameliorates *Escherichia coli* induced pneumonia

**Figure 4:**
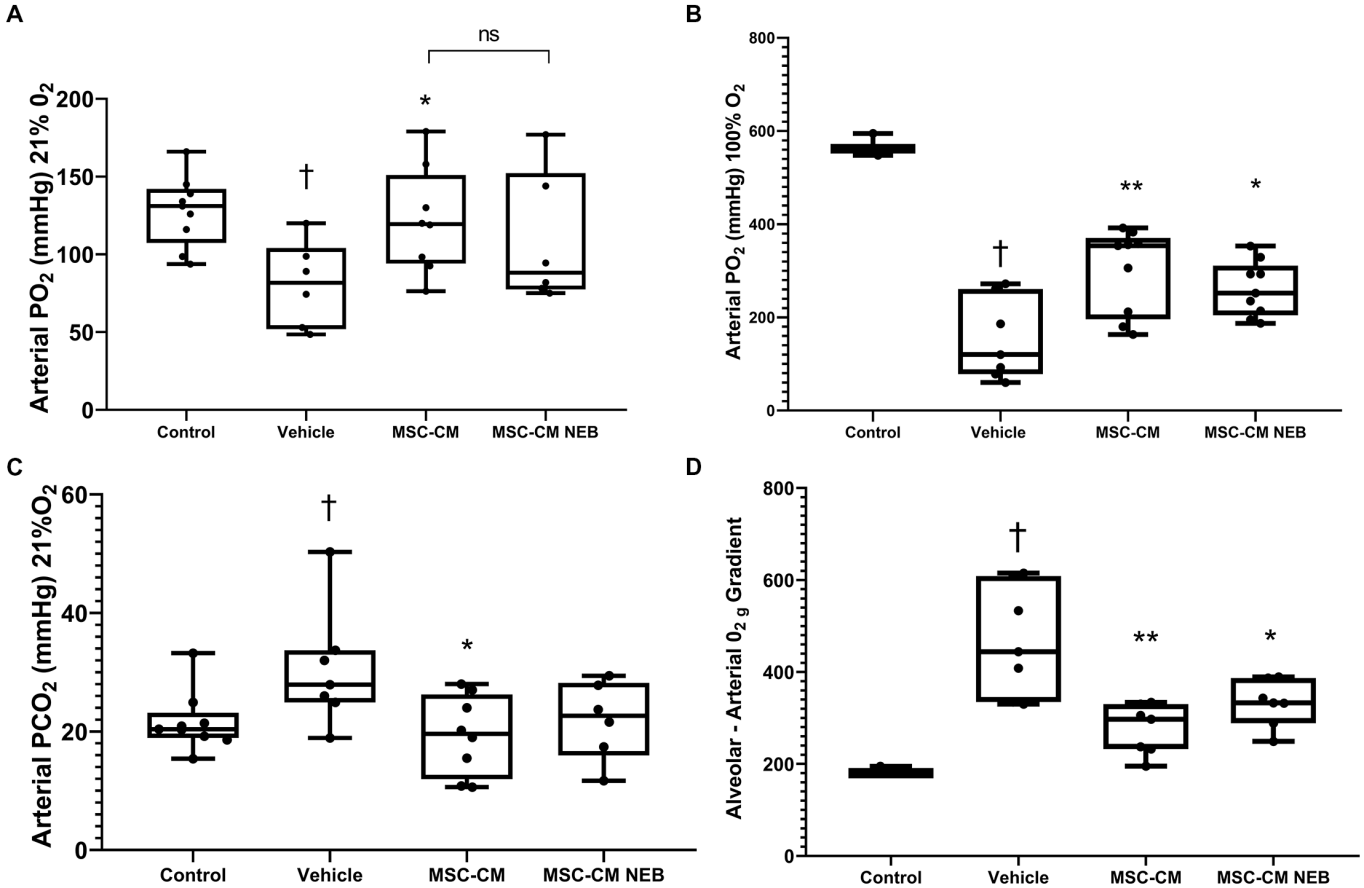
MSC-CM ameliorates physiological parameters in bacterial induced lung injury. Nebulization of MSC-CM 1 h after *Escherichia coli* installation mitigated the arterial PO_2_ decrease on 21% O_2_ **(A)** and 100% O_2_ **(B)** ventilation and attenuated PCO_2_ increase **(C)** at 48 h. No difference between delivery methods was observed. MSC-CM attenuated the increase in alveolar-arterial O_2_ gradient **(D)** with no statistical difference in effect based on the delivery methods (Data presented mean ± SD; ^†^significant between control and vehicle; ^*^significant difference between treated group and vehicle, both *p* ≤ 0.05; ^**^significant difference between treated group and vehicle *p* ≤ 0.01; and NS, no significant difference between nebulized and instilled delivery method).

**Figure 5:**
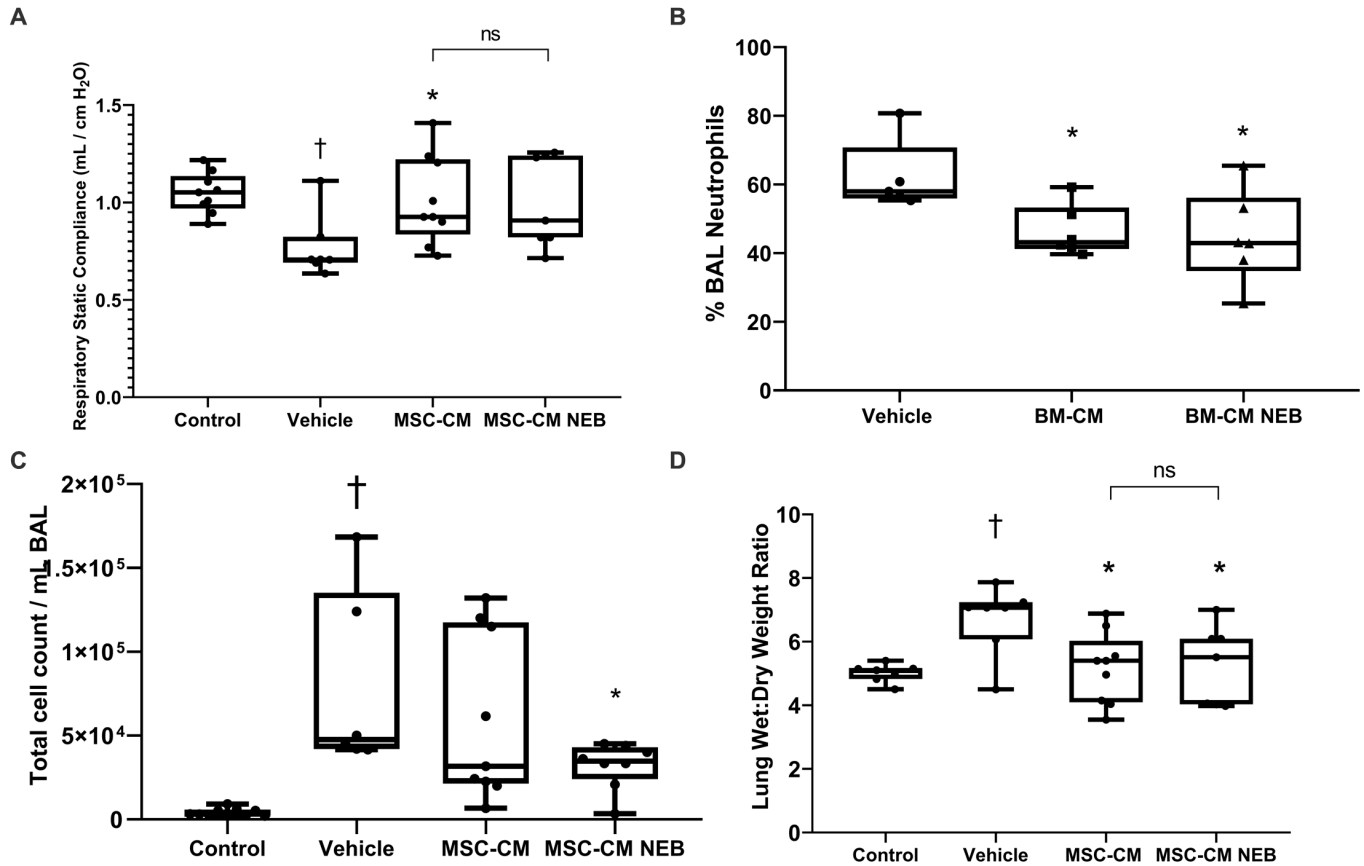
MSC-CM ameliorates other injury markers in bacterial induced lung injury. Nebulization of MSC-CM attenuated compliance decrease **(A)** while reducing total infiltrating cell count **(B)**, wet:dry ratio **(C)**, and neutrophils number **(D)** in the lung with no statistical difference between delivery methods (Data presented mean ± SD; ^†^significant between control and vehicle; ^*^significant difference between treated group and vehicle, both *p* ≤ 0.05; ^**^significant difference between treated group and vehicle *p* ≤ 0.01; and NS, no significant difference between nebulized and instilled delivery methods).

**Figure 6:**
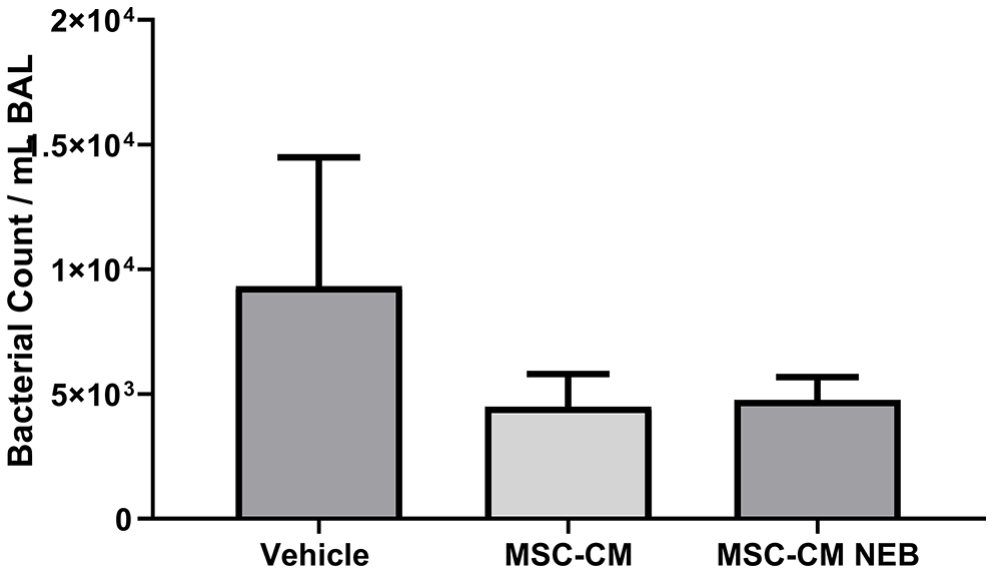
MSC-CM reduces bacterial load in bacterial induced lung injury. MSC-CM reduced the presence of *Escherichia coli* colony forming units in lung BAL measured 48 h after treatment with no difference between nebulized and non-nebulized CM (Data presented mean ± SD).

In a rodent model of pneumonia ARDS, instilled MSC-CM reduced the decrease in blood oxygenation observed in injured animals and this therapeutic effect could be reproduced with MSC-CM nebulized to the airspace IT (Figures 4A,B). This benefit did not reach significance for both modes at 21% inspired oxygen (Figure 4A) but did at 100% (Figure 4B). To further explore lung function parameters, blood CO_2_ levels were seen to rise with pneumonia injury compared to vehicle control, and this could be reversed by MSC-CM, with no significant difference between instilled or nebulizer delivery modes, however overall significance against vehicle was here lost with nebulization (Figure 4C). The calculated alveolar:arterial (A:a) gradient was also significantly improved by MSC-CM administered by either method (Figure 4D). Respiratory static compliance was improved by instilled MSC-CM alone (Figure 5A), but accumulation of fluid in the lung with pneumonia was reduced by MSC-CM delivered by instillation or nebulization (Figure 5B), an important parameter of lung function. With regard to infiltration of leukocytes to the airspace, significant numbers of total white cells accumulated in the BAL after pneumonia injury, and this was reduced by MSC-CM but only significantly so by direct IT instillation (Figure 5C). However, the percentage of these infiltrating cells that are of the important and more deleterious neutrophil type was significantly attenuated by both IT instilled and nebulized MSC-CM (Figure 5D). Finally, bacteria load in the BAL of animals with pneumonia injury was reduced by both instilled and nebulized MSC-CM although this did not reach significance in either modality (Figure 6).

### Nebulized MSC-CM reduced inflammatory cytokines in *Escherichia coli* induced pneumonia

**Figure 7:**
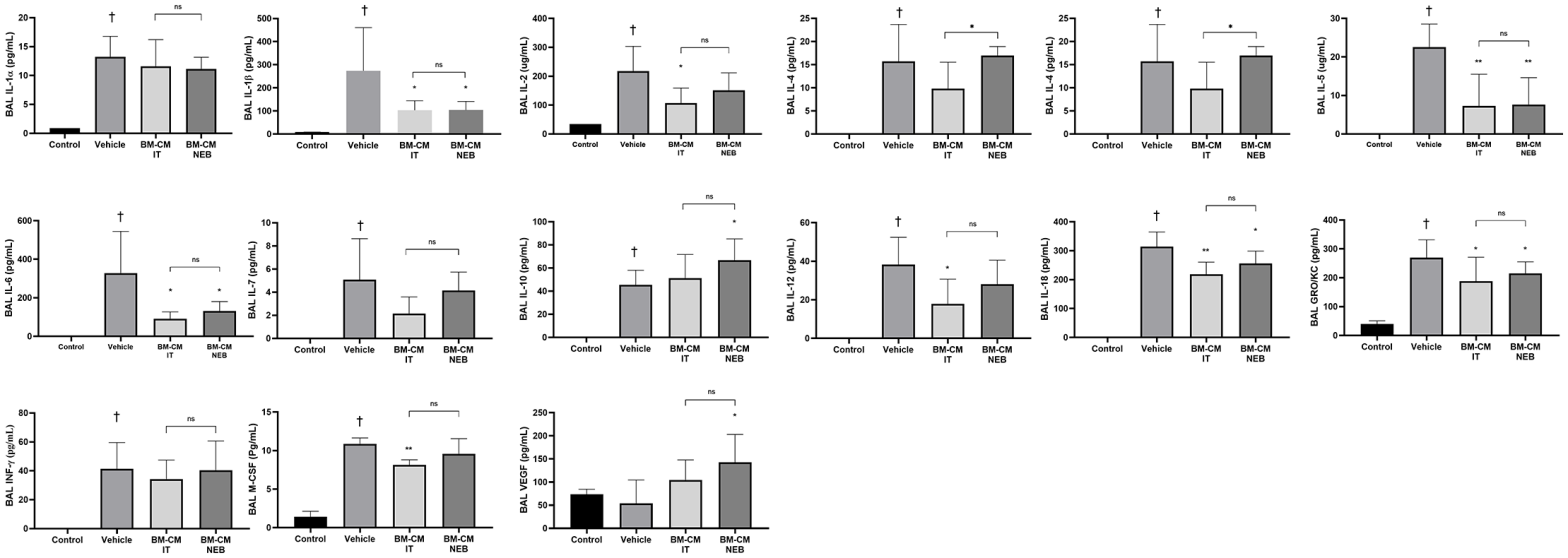
MSC-CM reduced inflammatory markers in lung after bacterial infection. Nebulizer administration of MSC-CM mitigated the increase of inflammatory cytokines IL-1α, IL-1β, IL-2, IL-4, IL-5, IL-6, IL-7, IL-12, IL-18, and IFN-γ while increasing anti-inflammatory cytokine IL-10 compared with the vehicle group at 48 h after injury induction with significant increase in effect with nebulised MSC-CM in IL-4 and IL-10, and no negative effect in the remaining cytokines. MSC-CM also mitigated the increase in chemoattractant GRO/KC and inflammatory related molecules such as M-CSF and VEGF after *Escherichia coli* administration 48 h after injury induction. No significance was observed between delivery routes (Data presented mean ± SD; ^†^statistically significant between control and vehicle group; ^*^significant difference between treated group and vehicle, *p* ≤ 0.05; and NS, no significant difference between nebulised and IT delivery route).

In a 23-plex cytokine assay, almost all cytokines measured were present in BAL in higher concentrations after *E. coli* pneumonia compared to healthy control animals (Figure 7). Cytokines reduced by MSC-CM instilled directly to the lung were: IL-1b, IL-2, IL-5, IL-6, IL-12. IL-18, GRO/KC, and M-CSF. Of these, all were also significantly ameliorated by IT nebulized MSC-CM apart from IL-2, IL-12, and M-CSF. The anti-inflammatory/pro-healing cytokines IL-10 and VEGF were both significantly increased by nebulized MSC-CM alone compared to vehicle control.

## Discussion

While there are promising preclinical findings and clinical trials are ongoing, there are still challenges associated with cell isolation, production, and administration that need to be solved in order for MSCs to become a feasible treatment for ARDS (10). MSC products are also gaining traction as therapeutics to replace the MSC itself, but these can be expensive and wasteful to isolate. Local administration of MSC conditioned media has been proven effective in the amelioration of lung injury after *E. coli* LPS endotoxin administration (21, 22). However, the intratracheal administration of MSC suspensions could produce a tissue injury due to the additional delivery of vehicle liquid to the lung, where nebulization can overcome local administration issues through delivery of a highly respirable aerosol containing the desired therapeutic over a longer time frame and with a deeper penetration throughout the lung tissue (23).

Supporting this idea, we have observed that nebulization of the MSC-CM allows penetration deep into the lung airways with a minimal waste of the product, confirming the compatibility of this delivery method with MSC cell products. In addition, the nebulization process did not alter the therapeutic capacity of the MSC-CM in reducing inflammatory cytokine concentrations and increasing cell viability after inflammatory damage *in vitro*. In the same vein, as McCarthy et al. (20) demonstrated recently, the direct antibacterial effects were also not adversely affected, with nebulized conditioned medium able to inhibit bacterial growth in live cultures of Gram-positive and Gram-negative bacteria species (20). Ionescu et al. demonstrated that IT delivered MSC-CM reduced the presence of neutrophils in the alveolar space in an LPS lung injury model (21). Similarly, we observed that IT delivery of MSC-CM reduced the immune cell presence in the lungs in a full *E. coli* pneumonia model, with a significant reduction in the percentage of neutrophils as reported in previous studies after whole cell administration (24). Interestingly, nebulization of the same MSC-CM product improved the reduction in infiltrating cells, particularly neutrophils, in the lungs compared with the IT delivery mode. In the same direction, MSC-CM produced a reduction in the fluid infiltration with a lower wet:dry weight ratio in treated animals regardless of the delivery methods. MSC-CM treated animals showed reduction in pivotal cytokines for the inflammatory process such as IL-1β and IL-6 similar to previous studies where whole MSCs were administrated (25). In addition, there were lower levels of immune cell chemoattractants like GRO/KC, possibly explaining the reduced presence of these cells in the lungs. There was also an increase in the levels of the cytokine IL-10, with an anti-inflammatory effect reminiscent of the findings reported by Gupta et al. after BM-MSC administration in an LPS lung injury model (14). The improvement observed in lung physiology translated into a better organ functionality as proven by the physiological parameters measured in the treated animals. Among these parameters, the alveolar-arterial gradient measurement is a strong indicator of gas exchange efficiency, with a high gradient relating to poor capacity for blood oxygenation (26). The reduction of the alveolar-arterial gradient, added to the reduction in arterial PCO_2_ and increase in the PO_2_, suggests a recovery of the blood-alveolar barrier and better O_2_/CO_2_ exchange in animals treated with nebulised MSC-CM.

As observed in previous studies, the MSC secretome modulates macrophage activity, generating an increase in M2 differentiated subpopulations, with anti-inflammatory cytokine production and increased phagocytosis (27). In this sense, we observed that nebulization of MSC-CM did not alter the capacity of MSC-CM to increase phagocytic activity in macrophages-like cells *in vitro*. This mechanism could be partially responsible for the reduction in bacterial presence observed in BAL samples from treated animals.

There are some limitations to this study. Firstly, transformed cell lines can only be considered the first line of *in vitro* investigation and deeper mechanistic studies should utilize primary human or animal lung epithelial cells. Secondly, the secretome is an incredibly complex mix of molecules and subcellular constructs, and determining which factors ultimately result in therapeutic effect is ongoing. The absence of a detailed mechanism of action may also have implications for potency assays during larger scale production and eventual trial licensing. Another limitation is that only a single timepoint of therapeutic delivery and injury assessment was possible within the scope of the study. Whether MSC-CM reduces the injurious process of ARDS or enhances the subsequent repair (or indeed both) is not answered. It also remains to be determined in MSC-CM can address a more established pneumonia infection and for the therapeutic window to be calculated.

In summary, the direct nebulization of MSC-CM broadly recapitulated the antibacterial and anti-inflammatory capacity of the MSC itself widely described in the scientific literature. In addition, nebulization enhanced the immune cell and fluid clearance capacity of the MSC-CM, offering a promising delivery route for the MSC secretome in ARDS patients where instillation of liquid therapeutics should be avoided.

## Data availability statement

The raw data supporting the conclusions of this article will be made available by the authors, without undue reservation.

## Ethics statement

The animal study was reviewed and approved by Animal Care in Research Ethics Committee (ACREC) at University of Galway.

## Author contributions

HEG: formal analysis, investigation, data curation, and writing—review and editing. SDM and CM: formal analysis, investigation, data curation, writing—review and editing, and supervision. JL: conceptualization, methodology, formal analysis, resources, writing—review and editing, and supervision. RM: conceptualization, methodology, formal analysis, investigation, resources, data curation, and writing—review and editing. DO’T: conceptualization, methodology, formal analysis, investigation, resources, data curation, writing—original draft, writing—review and editing, supervision, project administration, and funding acquisition. All authors contributed to the article and approved the submitted version.

## Funding

DO’T was supported by Health Research Board Ireland (ILP-POR-2017-024) and Science Foundation Ireland (13/RC/2073).

## Conflict of interest

RM is CSO of Aerogen Ltd.

The remaining authors declare that the research was conducted in the absence of any commercial or financial relationships that could be construed as a potential conflict of interest.

## Publisher’s note

All claims expressed in this article are solely those of the authors and do not necessarily represent those of their affiliated organizations, or those of the publisher, the editors and the reviewers. Any product that may be evaluated in this article, or claim that may be made by its manufacturer, is not guaranteed or endorsed by the publisher.
