# Supplementary material for: Nebulized mesenchymal stem cell derived conditioned medium ameliorates Escherichia coli induced pneumonia in a rat model
Source: Front Med (Lausanne). 2023 Jun 2;10:1162615. doi: 10.3389/fmed.2023.1162615 (PMC10272576; doi:10.3389/fmed.2023.1162615)
Supplement: Supplementary file 1 [file Data_Sheet_1.PDF]

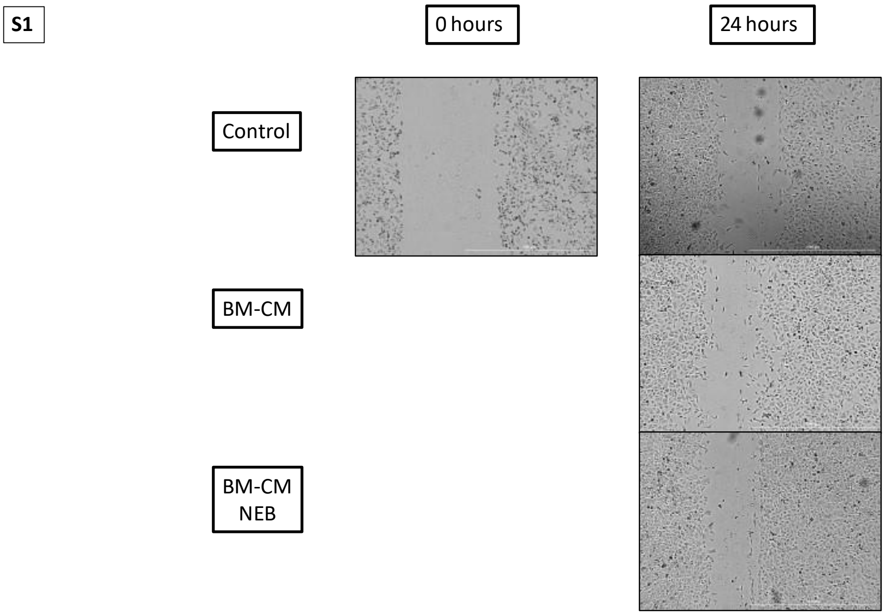

Supplementary Figure S1: Representative images of A549 monolayer scratch wound assay groups as presented in Figure 4F. 4x magnification.
